# Phytochemical and Pharmacological Study of the *Eysenhardtia* Genus

**DOI:** 10.3390/plants9091124

**Published:** 2020-08-31

**Authors:** Abraham Garcia-Campoy, Efrén Garcia, Alethia Muñiz-Ramirez

**Affiliations:** 1 Laboratorio de Investigación de Productos Naturales, Escuela Superior de Ingeniería Química e Industrias extractivas Instituto Politécnico Nacional, Av. Instituto Politécnico Nacional S/N, Unidad Profesional Adolfo López Mateos, Ciudad de Mexico CP 07708, Mexico; abrahamhgc27@hotmail.com; 2 Laboratorio de Química Supramolecular y Nanociencias, Instituto Politécnico Nacional, Acueducto S/N, Barrio la laguna Ticomán, Ciudad de Mexico CP 07340, Mexico; efren1003@yahoo.com.mx; 3 CONACYT-IPICYT/CIIDZA, Camino a la Presa de San José 2055, Col. Lomas 4 Sección, San Luis Potosí S.L.P CP 78216, Mexico

**Keywords:** *Eysenhardtia*, phytochemical, pharmacological

## Abstract

The participation of natural products in health care has been remarkable, and today they continue to play a key role in the discovery and development of new treatments. Phytochemical studies together with pharmacological tests have managed to integrate bioactive agents as an alternative solution to reduce or regulate the problems caused by diseases. The *Eysenhardtia* genus is a family of plants that are rich in secondary metabolites, which have shown potential activity in the control and mitigation of urinary disorders, diabetes, oxidative stress, protein glycosylation, microbial infections, inflammation, pain or discomfort, muscle contractions, cytotoxicity, or as a cellular or neuronal signaling modulator. These conditions generally appear in comorbid diseases, which motivated the bibliographic review associated with the plant. This document presents the beneficial actions produced by *Eysenhardtia* extracts and/or bioactives to inhibit, control, or reduce the complications or discomfort of degenerative diseases and thus generate new therapeutic alternatives.

## 1. Introduction

The relationship between humanity and medicinal plants goes back to ancient times. The oldest records found correspond to the Sumerians, approximately 5000 years ago [1]. The growth and development of civilizations have been closely linked to the use and exploitation of the healing properties of medicinal plants [2]. The process of recognition and use of herbalism in the preparation of remedies, infusions, or concoctions for the treatment of disease has been carried out by trial and error [3]. There are countries with a long tradition of using herbal medicine such as China, India, Japan, Pakistan, and Mexico. Ethnopharmacological studies have contributed to the amplification of knowledge and the discovery of new drugs or effective bioactive compounds for the control of diseases [4].

Mexico has a history of using ethnomedicine, whose origins go back to pre-Hispanic cultures where archaeological findings have shown its anthropological influence and impact on Mexican culture [5]. One of the plants with antecedents is *Eysenhardtia polystachya* (EP), dating back to the era of New Spain where Nicolas B. Monardes (1565) relates the use of a tree to treat kidney and urinary diseases; he describes that they drank a blue-colored infusion which was obtained by placing thin slices of a wood (bark) into water for a period of time [6]. Furthermore, in the Florentine Codex, Fray Bernardino de Sahagún refers to the use of “Coatli” for fever or retention of urine [7]. In the following centuries (XVI and XVII), it was taken to Europe where it was known as *Lignum nephriticum* [8,9]. Later, it continued to be consumed in a traditional way by Mexicans for the control of urinary, contraceptive [10], antidiarrheal, and antiseptic disorders, as well as for the control of diabetes [11,12].

In recent decades, the *Eysenhardtia* genus has attracted attention due to its medicinal properties. This triggered a series of phytochemical and pharmacological studies linked to the identification of health benefits. Among the actions determined are its diuretic [13], antidiabetic, antiglycation [14], antioxidant [15], anti-inflammatory [16], and antimicrobial [17] potential. It also has cytotoxic properties [8], is cardioprotective, and inhibits neurodegeneration and modulators of cell signaling [15].

This article presents a bibliographic review focused on the diverse activities that have been attributed to the *Eysenhardtia* genus, which is made up of fourteen species [18].

## 2. Taxonomic Classification

Kingdom: Plantae

Phyllum: *Tracheophyta*

Class: *Magnoliopsida*

Orden: *Fabales*

Family: *Fabaceae*

Species: *E. adenostylis, E. platycarpa, E. punctata, E. subcoriacea, E. cobriformis, E. reticulata, E. orthocarpa, E. polystachya, E. texana, E. angustifolia, E. spinosa, E. peninsularis, E. parvifolia, E. schizocalyx*.

Common names in Mexico: Cuate (Jal.), Coatillo (Pue.), Coatl (l. náhuatl), Cohuatli, Cuatle (Oax.), Lanaé (l. chontalpa, Oax.), Palo cuate, Rosilla (Sin.), Palo dulce (Sin., Mex., Hgo., Pue., Mich.), Palo azul (Mex), Taray (N.L., Dgo.), Tlapahuaxpatli; Ursa (l. otomí, Hgo.), Vara dulce, Varaduz (Dgo.).

Common names in English: Kidneywood, Mexican Kidneywood [19,20,21].

### 2.1. Description of the Eysenhardtia Genus

**Figure 1:**
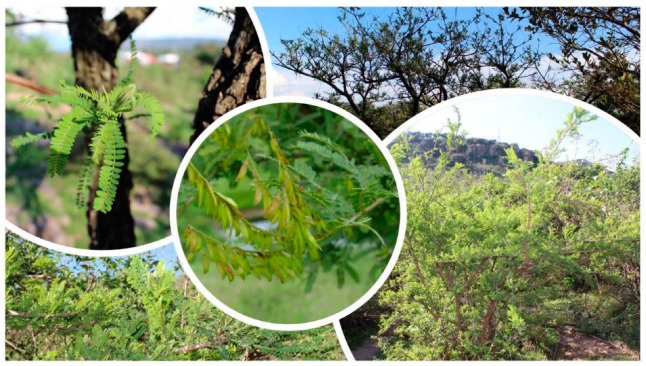
*Eysenhardtia* genus.

The *Eysenhardtia* genus is a deciduous tree or shrub 3 to 9 m high with a diameter of 15 to 35 cm [20]. It has a crown and alternate, compound, pinnate leaves, elliptical leaflets, and aromatic resins. Its wood is branched brown [19]. The bark is rough and scaly with a dark coloration on the outside and reddish-brown on the inside [20]. It shows inflorescences in spiky clusters lobed with white corolla. Its fruit is shown in a curved pod that houses a thin seed susceptible to water, and it is a hermaphrodite (Figure 1) [17].

### 2.2. Geographical Distribution of the Eysenhardtia Genus

**Figure 2:**
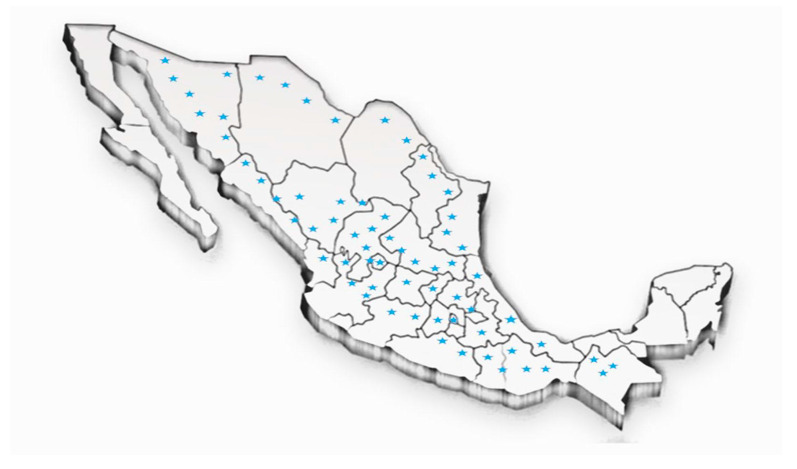
Geographical distribution of *Eysenhardtia* [22,23].

It is found throughout Mexico, mainly in the states of Colima, Chiapas, Chihuahua, Coahuila, Mexico City, Durango, Guanajuato, Guerrero, Hidalgo, Jalisco, State of Mexico, Morelos, Oaxaca, Puebla, Querétaro, San Luis Potosí, Sinaloa, Sonora, Tamaulipas, Tlaxcala, Veracruz, and Zacatecas. It has also been found in the southeastern United States (Figure 2) [22,23].

## 3. Secondary Metabolites Isolated from the *Eysenhardtia* Genus

The *Eysenhardtia* genus has shown to be an excellent source of secondary metabolites with flavonoids, flavones, isoflavones, flavonones, phenolic compounds, chalcones, dihydrochalcones, coumarins, pterocarpan, sugars, and fatty acids, among others, in its composition. Table 1 shows the phytochemical compounds identified from the different parts (leaves, branches, heartwood, and bark) of the genus.

## 4. Pharmacological Activities

The pharmacological activities were described chronologically in order to encapsulate the research carried out on *Eysenhardtia* gender through the years.

### 4.1. Urinary Disorders

As previously mentioned, the plant was analyzed for its beneficial effects on kidney or urinary tract problems. In 1998 carried out a study to test the diuretic and antilytic effect of the aqueous extract of *Eysenhardtia polystachya* bark in rats [37]. The model animals were induced with urolithiasis by implanting a zinc disk in the bladder. As a result, they observed a significant reduction in the weight of the uroliths after the administration of the aqueous extract of *E. polystachya* in the affected animals compared to the control group. Diuretic action was also verified by identifying an increase in the volume of urine [37], as well as favoring the retention of potassium [38].

In the year 2000, the compounds responsible for the antiurolithiatic (antilithiatic) and diuretic activity were identified, which correspond to 7-hydroxy-2′,4′,5′-trimethoxyisoflavone (16) and 7-hydroxy-4′-methoxyisoflavone (23) [28,39].

Later, in 2002, the effect of isoflavones isolated from the bark of *Eysenhardtia polystachya* on the formation of oxalate and calcium phosphate (COM) stones in urine was analyzed and reported in a preclinical trial. From this study, it was found that the phenolics compounds reported were able to inhibit the formation and growth of COM, consequently reducing the appearance of kidney stones and suggesting the use of isoflavones as a preventive treatment [29].

### 4.2. Antimicrobial Activity

The inappropriate use of treatments and the ability of microorganisms to adapt have led them to develop tolerance to various antimicrobial drugs; this has prompted the continued search for new drugs that offer other options, possible sources thereof being plants. In the case of the genus Eysenhardtia, the first report found on antimicrobial activity was published in 1997, in which an antimicrobial scan was performed using minimal inhibitory concentration (MIC) tests. The microorganisms used were *Sarcina lutea, Proteus vulgaris, Staphylococcus aureus, Escherichia coli,* and *Candida albicans*. The information obtained showed that the *E. polystachya* methanol extract had an effect on inhibiting the different strains studied; this inhibitory action was shown to have potential against Gram positive and Gram negative bacteria [40].

In 1998, the antimicrobial action of the compounds (3*S*)-7-hydroxy-2′,3′,4′,5′,8-pentamethoxyisoflavan (10), (3*S*)-3′,7-dihydroxy-2′,4′,5′,8-tetramethoxyisoflavan (11), stigmasterol (68), isoduartin (14), cuneatin (15), 7-hydroxy-2′,4′,5′-trimethoxyisoflavone (16) and 3,4-dimethoxy-8,9-methylenedioxypterocarpan (7), isolated from the bark *E. polystachya* was analyzed for each of it. The results showed that they had no action against *Escherichia coli, Pseudomonas aeroginosa, Streptococcus aureus, Bacillus subtilis, Shigella smiled*, and *Candida albicans* at a concentration of 200 µg/mL [8].

In 1999, the compounds (*α*-hydroxydihydrochalcones, (*αR*)-*α*,3,4,2′,4′-pentahydroxydihydrochalcone (17), (*αR*)-3′-C-*β*-D-xylopyranosyl-*α*,3,4,2′,4′-pentahydroxydihydrochalcone (18), (*αR*)-3′-*O*-*β*-D-xylopyranosyl-*α*,3,4,2′,4′-pentahydroxydihydrochalcone (19) and coatline B [(*αR*)-3′-C*-β*-D-glucopyranosyl-*α*,2′,3,4′,4-pentahydroxydihydrochalcone]) (2) isolated from the bark of *E. polystachya*. They did not show activity against *Escherichia coli*, *Pseudomonas aeroginosa*, *Streptococcus aureus*, *Bacillus subtilis*, *Shigella smiled*, and *Candida albicans* [25]. Likewise, in 1999 were evaluated the compounds 4′,5,7-trihydroxy-8-methyl-6-(3-methyl-[2-butenyl])-(2*S*)-flavanone (20), 4′,5,7-trihydroxy-6-methyl-8-(3-methyl-[2-butenyl])-(2*S*)-flavanone (21) and 4′,5-dihydroxy-7-methoxy-6-(3-methyl-[2-butenyl])-(2*S*)-flavanone (22), from the aerial zone of *Eysenhardtia texana Kunth* from an extract of methanol and dichloromethane. The flavonones were active in inhibiting the growth of *Staphylococcus aureus*, and one of them also reduced the proliferation of *Candida albicans* [26,27].

Subsequently, the antimicrobial action of the methanol extracts of *E. polystachya* and *E texana* were studied to mitigate nine strains of bacteria and a yeast linked to urinary infections; the species evaluated were *Escherichia coli*, *Proteus mirabilis*, *Proteus vulgaris*, *Klebsiella pneumoniae*, *Enterobacter aerogenes*, *Pseudomonas aeruginosa*, *Serratia marcescens*, *Staphylococcus aureus*, *Staphylococcus epidermidis*, and *Candida albicans*. The results showed that *E. texana* had limited antibacterial activity; in contrast, *E. polystachya* showed a broad-spectrum antimicrobial effect against the microorganisms evaluated. The authors suggest its use as a possible alternative for the inhibition of Gram positive and Gram negative bacteria, since it exists there is a shortlist of appropriate drugs to inhibit these families of microorganisms [18].

In 2012, the antibacterial potential of 47 plants in oral infections was researched by analyzing ethanolic and aqueous extracts. *E. polystachya,* showed a reduction in the proliferation of *Streptococcus mutans* and *Porphyromonas gingivalis* [41].

Subsequently, the ethanol extract from the leaves and branches of *E. polystachya* was analyzed using the MTT test, as well as the lowest inhibitory concentration (MIC). The data showed activity for *Escherichia coli* (MIC = 1.56 µg/mL), *Staphylococcus aureus* (MIC = 0.78 µg/mL), and multi-resistant organisms (MIC > 100 µg/mL) [12].

### 4.3. Antidiabetic Activity

Diabetes is a chronic degenerative disease related to hyperglycemia. Excess glucose in the blood causes health problems related to the generation of reactive oxidant species, the glycation of proteins, lipoperoxidation, difficulty in recognizing, sensitivity, and secretion of insulin, among others; this results in a diabetic pathology.

In 1998, the hypoglycemic capacity of 28 plants was evaluated; among the species analyzed was *E. polystachya*, which was considered an appropriate candidate to regulate glycemic levels in a pre-clinical study [11].

Subsequently, the antihyperglycemic activity of the methanolic extracts of the leaves (LEP), branches (BEP), and bark (REP) of *Eysenhardtia platycarpa* was studied in diabetic rats induced with streptozotocin, as well as in healthy animals. It was observed that the REP extract did not exhibit antihyperglycemic action compared to LEP and BEP. Additionally, the effect of 3-*O*-acetyloleanolic (72) acid (isolated from BEP) was observed in reducing blood glucose levels in diabetic rats.

In 2007, a study was published evaluating the antioxidant activity of the methanol extracts of *E. platycarpa, E. puntacta,* and *E. subcoriacea* in a murine pancreatic model. The tests showed that these species were protected from the damage induced by 2,2-azo-bis(2-amidinopropane)dihydrochloride (AAPH). Furthermore, some bioactives, (3*-O*-acetyl-11*α*,12*α*-epoxy-oleanan-28,13*β*-olide (69), (+)-catechin (33), and (+)-catechin-3-*O-β*-D-galactopyranoside (37)) isolated from the branches of *E. platycarpa*, showed radical scavenging capacity 2,2-diphenyl-1-picrylhydrazyl (DPPH), increase the concentration of pancreatic glutathione (GSH), the activity of glutathione peroxidase (GSHPx) and catalase (CAT), as well as regulating the gain in levels of glucose in diabetic rats induced with streptozotocin after five days of administration [33].

In 2008, the antioxidant activity of secondary metabolites identified in the aerial zone of *E. subcoriacea* was reported. Among the bioactive drugs described are 3-(2′-hydroxy-4′,5′-methylendioxyphenyl)-6-(3′’-hydroxymethyl-4′’-hydroxybut-2′’-enyl)-7-hydroxycoumarin (32), (+)-catechin (33), (-)-epicatechin (34), (+)-afzelechin (35), eriodictyol (36), (+)-catechin 3-*O-β*-D-galactopyranoside (37), and quercetin 3-*O-β*-D-galactopyranoside (38), all of which countered the presence and development of free radicals (DPPH and AAPH) and can be used to suppress diabetic pathology [31].

In 2010, the antihyperglycemic and antioxidant effect of subcoriacin (3-aryl-6prenylcoumarin), a secondary metabolite from *E subcoriacea*, was researched. The administration of the bioactive compound to streptozotocin-induced diabetic rats for five days showed a reduction in blood glucose levels, increased activity of the enzymes (glutathione peroxidase (GSHPx), superoxide dismutase (SOD) and catalase (CAT)) of the endogenous system. For this reason, the protective action of Subcoriacin against pancreatic damage induced by free radicals under a diabetic condition [32] was suggested.

In 2014, the antioxidant, antidiabetic and antiglycation properties of the aqueous-methanolic extract of *Eysenhardtia polystachya* were analyzed. The ability to decrease and/or eliminate free radicals DPPH, ABTS, was noted, as well as the reactive oxygen species (RO_2_, −O_2_, H_2_O_2_, −OH, ONOO−, NO−, HOCl, ^1^O_2_), and the ability to absorb oxygen radicals (ORAC) by donating electrons (H^+^). The ability to sequester metals for the formation of chelates and decreased lipoperoxidation was also examined. At the same time, the activity to inhibit fluorescent AGEs, glycation of hemoglobin, and methylglyoxal products was determined. As for in vivo tests with strepzotocin-induced diabetic murine models, a decrease was observed in blood glucose levels, triglycerides (TG), total cholesterol (TC), thiobarbituric acid reactives substances (TBARS), and low-density lipoprotein (LDL). At the same time, an increase in serum insulin, body weight, and liver biomarkers were induced. Consequently, the methanolic-aqueous extract of EP demonstrated the ability to interact at the different points evaluated, exercising beneficial actions in the control of disorders associated with diabetes based on the presence of phenolic compounds [14].

In 2016, were analyzed six compounds (2′,4′-dihydroxychalcone-6′-*O-β*-D-glucopyranoside (39), *α*,3,2′,4′-tetrahydroxy-4-methoxy-dihydrochalcone-3′-C-*β*-glucopyranosi-6′-*O*-*β*-D-glucopyranoside (40), 7-hydroxy-5,8′-dimethoxy-6′*α*-L-rhamnopyranosyl-8-(3-phenyl-transacryloyl)-1-benzopyran-2-one (41), 6′7-dihydroxy-5,8-dimethoxy-8(3-phenyl-trans-acryloyl)-1-benzopyran-2-one (42), 9-hydroxy-3,8-dimethoxy-4-prenylterocarpan (43), and *α*,4,4′-trihydroxy-dihydrochalcone-2′-*O-β*-D-glucopyranoside (45)) from the aqueous-methanol extract of the bark of *Eysenhardtia polystachya*, for their possible antioxidant potential, in streptozotocin-induced diabetic mice. The first five flavonoids showed favorable effects on the regulation and protection of hepatic and cellular biomarkers associated with disorders caused by oxidative stress in diabetes [34].

Furthermore, the inhibition capacity of 11 dihydrochalcones from the methanolic extract of the *E. polystachya* bark in the formation of advanced glycation end products (AGEs) was studied in their multiple stages (early, intermediate, and final). Protein glycation corresponds to the interaction of proteins with excess sugars in the body; this hyperglycemic condition occurs during diabetes. The tests carried out were in vitro, and it was concluded that each of the isolated bioactives were capable of inhibiting and protecting from the formation and accumulation of AGEs. The biocomposites analyzed were: (6′methoxy-sieboldin (53), 2′-*O*-*α*-L-rhamnopyranosyl-*α*,6′,dihydroxy-4′acetyl-4-methoxy-dihydrochalcone (52), 2′-*O-β*-D-glucopyranosyl-4′-methoxy-4-hydroxy-3-isoprenyl-dihydrochalcone (54), 2′,4′,6′trihydroxy-4,5-dimethoxy-3-isoprenyl-dihydrochalcone (55), 3′-C-*β*-glucopyranosyl-*α*,2′,4′,6′-trihydroxy-4-methoxy-dihydrochalcone (56) and 3′-C-*β*-glucopyranosyl-*α*,2′,4-trihydroxy-4′,6′-dimethoxy-dihydrochalcone) (57) the rest (3-hydroxyphloretin-4′-*O-β*-D-glucopyranoside (58), 3,4′-dihydroxy-2,4,6-trimethoxy-dihydrochalcone (59), 2′,4′,4-trihydroxy-3′-methoxy-dihydrochalcone (60), 2′,4′,6′,4-tetrahydroxy-3,5-diisoprenyl-dihydrochalcone (61), and 3′-C-*β*-glucopyranosyl-*α*,2′,4′,3,4-penta-hydroxy-dihydroxychalcone (62)) had previously been elucidated [35].

In 2019, it was demonstrated that the bioactive 3′-*O*-*β*-D-glucopyranosyl *α*,4,2′,4′,6′-pentahydroxy–dihydrochalcone (63) isolated from *Eysenhardtia polystachya* is an excellent antiglycation compound capable of reducing glycation of proteins responsible for diabetic nephropathy in diabetic individuals. Tests were carried out using diabetic mice with kidney problems induced with stretozotocin; levels of glycated hemoglobin (HbA1c), the concentration of glycation end products advances in the kidney and circulatory system, as well as in pro-inflammatory markers ICAM-1 were then analyzed. The bioactive (63) was administered for five weeks at different concentrations (25, 50, and 100 mg/kg); at the end of the dosing period the diabetic animals showed a significant improvement, suggesting the ability of the bioactive to react and inhibit early and intermediate precursors in the formation of AGEs, responsible for damaging the structure of the kidneys [36].

### 4.4. Anti-Inflammatory Activity

In 2015, the anti-inflammatory potential of the *Eysenhardtia polystachya* bark from hexane (PAH), chloroform (PAC), and methanol (PAM) extracts was researched. The extracts were evaluated using Wistar rats as an experimental model. To determine the anti-inflammatory activity of the extracts, different tests were used such as: carrageenan and croton oil induced edema. The tumor necrosis factor *α* (TNF-*α*), interleukin-1-*β* (1L-1*β*), prostaglandin E2 (PGE2) and leukotriene B4 (LTB4) were also quantified. The PAM extract showed anti-inflammatory activity in the different induced edema inhibited the expression of the cytokines (TNF-*α*, 1L-1*β*, PGE2 and LTB4), and the enzymes lipoxygenase and xanthine-oxidase linked to inflammatory problems [15].

Furthermore, in 2018 the properties of the ethanolic extract from the bark of *E. polystachya* to mitigate rheumatoid arthritis were analyzed. To do this, the antiarthritic activity induced by Complete Freund’s Adjuvant (CFA) in rats was determined. Trials showed that secondary metabolites of *E. polystachya*, mainly flavonoids, inhibited secondary inflammatory processes in arthritic rats, encouraged histopathological changes, and reduced serum levels of inflammatory cytokines [16].

Additionally, in that same year, the anti-inflammatory effect of ethanol extract from the leaves and branches of *E. polystachya* was examined using in vitro tests stimulating macrophages using LPS. The results demonstrated a decrease in the production of H_2_O_2_ (IC_50_ = 43.9 ± 3.8 µg/mL) and IL-6 (73.3 ± 6.9 µg/mL) [12].

### 4.5. Antinociceptive Activity

The importance of reducing or mitigating the sensation of pain has been fundamental for the control and treatment of different conditions, this has prompted the search for natural alternatives. In 2018, the ethanolic extract of the bark of *E. polystachya* was evaluated, using the acetic acid-induced abdominal contraction test and the hot-plate test, which were performed on mice. A decrease in the spasms generated by acetic acid and a prolonged sensitivity time at the peripheral and central levels [16] were observed.

Furthermore, in that same year, D-pinitol (80) isolated from ethanolic extract of *Eysenhardia polystachya* was examined to find out if it had the property to block or reduce pain. It was found that D-pinitol inhibited 67.58% with an effective dose of 10.7 mg/kg; its activity is associated with the serotoninergic system (5-HT3) and nitric oxide [42].

Additionally, the antinociceptive effect of the ethanolic extract from leaves and branches of *E. polystachya* (EPE) was also studied, following the acetic acid, formalin, and hot plate test. It was identified that the extract contained 26.93% D-pinitol, the bioactive responsible for an action. EPE presented an effective dose of 117 ± 14.5 mg/kg and 33 ± 3.2 mg/kg for D-pinitol in acetic acid test; in the case of formalin it was 48.9 ± 3.9 mg/kg. The action of EPE is probably due to the involvement of ATP-sensitive K+ channels. As for D-pinitol, its route of action is considered to occur at nitric oxide receptors and 5-hydroxytryptamine 3 (5-HT3) [12].

### 4.6. Antidiarrheal

The ability to stop diarrhea was analyzed from the ethanolic extract of the leaves and branches of *E. polystachya* (EPE) and the compound D-pinitol (80) isolated from *E. polystachya*. The results showed that the EPE extract had an effective dose of 7.5 ± 0.9 µg/mL and the D-pinitol 0.1 ± 0.03 µg/mL. The results suggest that this was due to the elimination of intestinal fluid [12].

### 4.7. Muscle Relaxant

The pharmacological potential in Jejunum muscle contraction was explored using chloroform extract from the stems of *E. polystachya* in muscle tissue extracted from rabbits. The test showed that the extract presents an effect on jejunum contractility [43].

### 4.8. Cytotoxic Activity

In 1998, the cytotoxic activity of the bioactives ((3*S*) -7-hydroxy-2′,3′,4′,5′,8-pentamethoxyisoflavan (10), (3*S*)-3′,7-dihydroxy-2′,4′,5′,8-tetramethoxyisoflavan (11), isoduartine (2′,7-dihydroxy-3′,4′,8-trimethoxyisoflavan)(14)) was evaluated; these were isolated from the chloroform-methanol extract of the bark of *E. polystachya*. The identified metabolites showed slight cytotoxic activity against the cell lines (KB (nasopharyngeal carcinoma), P388 (murine leukemia) and SQC-1 UISO (squamous cell carcinoma of the cervix)) [8].

### 4.9. Nanoparticles and Eysenhardtia

Furthermore, in 2018 silver nanoparticles were synthesized with the aqueous methanol extract of *Eysendhartia polystachya* and their performance in inhibiting AGEs in vitro tests cell viability with RAW-264.7 cells was analyzed and in vivo tests with diabetic zebrafish induced by glucose exposure were performed. It was found that the bioactives present in the nanostructures can counteract protein glycosylation through their intervention in the neutralization and elimination of fluorescent, non-fluorescent, free radical AGEs, and the catchment of methylglyoxal together with its derivatives. Regarding diabetic fish, these showed a decrease in the conditions caused by chronic hyperglycemia. Their results suggest the use of nanoparticles for diabetic pathology [44].

Also, in 2018, silver nanoparticles biosynthesized with the aqueous methanolic extract from the bark of *E. polystachya* were used as delivery and transport systems for polyphenolic constituents. The cell viability (murine RAW-264.7 macrophage cells) and biocompatibility of nanostructures was determined, as well as the *E. polystachya* extract. The antioxidant effect of the synthesized nanometric material on the biomarkers of zebrafish embryos exposed to a medium with high glucose concentrations was also studied. Treatments using nanoparticles and plant extracts increased the activity of the antioxidant enzymes (SOD, CAT, and GPx) and reduced the damage caused by the formation of reactive oxidative species, positively influencing the total protein concentration while decreasing the formation of malondialdehyde (MDA) and lipoperoxidation. Consequently, nanostructures with a high content of phenolic compounds could be a viable alternative in minimizing the diabetic complications associated with oxidative stress [45].

In that same year, the antidiabetic properties (qualities) of the bio-nanofabricated silver nanoparticles were evaluated using the hydroalcoholic extract of *Eysenhardtia polystachya* (EP/AgNPs). They demonstrated the protective action of EP/AgNPs against damage caused by hydrogen peroxide to INS-I cells, minimizing cell mortality, also having a cytoprotective action against oxidative laceration. Furthermore, it was observed that when administering the EP/AgNPs to glucose-induced diabetic zebrafish (111 mM), they showed an improvement in the levels of blood glucose, insulin, triglycerides, and cholesterol; helping to regulate hyperglycemic, hyperlipidemic, and insulin sensitivity. The authors suggest the possibility of using *E. polystachya* nanostructures as an alternative therapy for the control of diabetes due to their high concentration of chalcones, dihydrochalcones, and flavonoids [46].

Furthermore, nanoparticles have been synthesized and functionalized with the extract of *E. polystachya* using biological applications by taking advantage of the composition of the plant’s fluorescent biocomposites; these can then be used as a fluorescent cell nanomarker to detect cancer or pathogenic microorganisms [47].

### 4.10. Other Applications

The bioactive molecules responsible for the fluorescence of *E. polystachya* were isolated and elucidated from the methanol extract of the heartwood. Among the bioactives isolated are; 7-hydroxy-2′,4′,5′-trimethoxyisoflavone (16) and 9-methoxy-2,3-methylenedioxycoumestan (47) corresponding to Robert Boyle’s acid-base fluorescent indicator used in the 17th century [24].

Also, the activity of the extracts of ethyl acetate, dichloromethane, hexane, and methanol was determined from calluses and suspended cells of *E. polystachya* for the control of fungal phytopathogens. The information obtained provided an opportunity to propose plant extracts as a fungicide option [48].

Nine fractions of the *E. polystachya* ethanolic extract were also evaluated for use as fluorescent biosensors. Six of the nine fractions tested positive for photoluminescence and only two were stable for use in the functionalization of silica nanoparticles. The G2 subfraction demonstrated appropriate fluorescence qualities at a physiological pH and its detection also did not show toxicity, allowing cell viability [49].

## 5. Conclusions

Currently, there are many diseases linked to factors related to metabolic syndrome. This condition has caused people to develop various health problems and increases their risk of mortality. This has caused a large part of society to live under a constant medication scheme that attempts to regulate the ailments that afflict them, which in turn stimulates the consumption of natural alternatives that have shown to help control symptoms. In this paper, we present the potential of the extracts and/or bioactives of the *Eysenhardtia* genus to limit the proliferation of microbial infections, urinary disorders, oxidative stress, protein glycation, lipoperoxidation, increase in blood sugar levels, inflammation, or the development of bodily discomfort. Furthermore, the introspection performed made it clear that the composition of phytochemical compounds and their diversity of secondary metabolites could be a complementary solution for individuals affected by comorbidity.

## Figures and Tables

**Table 1:** Phytochemical compounds identified in the species *Eysenhardtia*.

Phenolic Compounds					
Number	Compound	Structure	Species	Part Used (Type of Extract)	Reference(s)
**1**	coatline A (3′*-C-β*-glucopyranosyl-*α*,2′,4′,4-tetrahydroxydihydrochalcone)	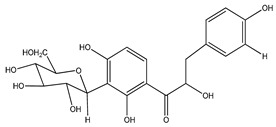	*E. polystachya*	Wood (aqueous)	[13]
**2**	coatline B (3′-*C-β*-glucopyranosyl-*α*,2′,4′,3,4-pentahydroxydihydrochalcone)	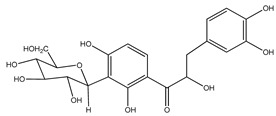	*E. polystachya*	Wood (aqueous)	[13]
**3**	matlaline	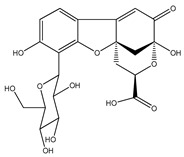	*E. polystachya*	Wood (aqueous)	[7]
**4**	octa-acetyl coatline A	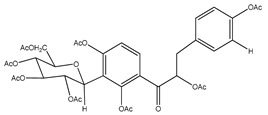	*E. polystachya*	Wood (aqueous)	[13]
**5**	nona-acetyl coatline B	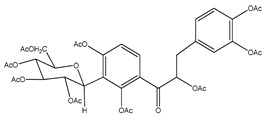	*E. polystachya*	Wood (aqueous)	[13]
**6**	nona-acetyldihydrocoatline B	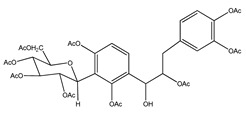	*E. polystachya*	Wood (aqueous)	[13]
**7**	3,4-dimethoxy-8,9-methylenedioxypterocarpan	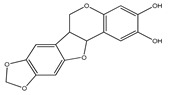	*E. polystachya*	Heartwood (methanol) Bark (CHCl_3_-MeOH)	[8,24]
**8**	dehydrorotenone	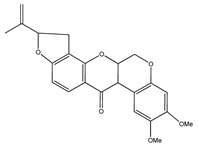	*E. polystachya*	Heartwood (methanol)	[24]
**9**	angustlegor-retoside	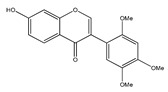	*E. polystachya*	Heartwood (Methanol)	[24]
**10**	(3*S*)-7-hydroxy-2′,3′,4′,5′,8-pentamethoxyisoflavan	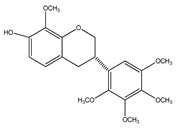	*E. polystachya*	Bark (CHCl_3_-MeOH)	[8]
**11**	(3*S*)-3′,7-dihydroxy-2′,4′,5′,8-tetramethoxyisoflavan	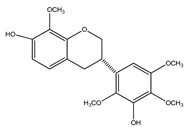	*E. polystachya*	Bark (CHCl_3_-MeOH)	[8]
**12**	(3*S*)-2′,3′,4′,5′,8-pentamethoxy-7-*O*-acetylisoflavan	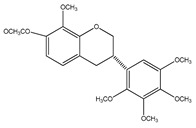	*E. polystachya*	Bark (CHCl_3_-MeOH)	[8]
**13**	(3*S*)-2′,4′,5′,8-tetramethoxy-3′,7-*O*-diacetylisoflavan	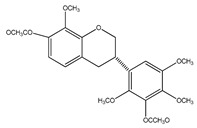	*E. polystachya*	Bark (CHCl_3_-MeOH)	[8]
**14**	isoduartin (2′,7-dihydroxy-3′,4′,8-trimethoxyisoflavan)	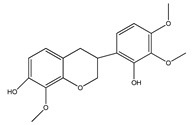	*E. polystachya*	Bark (CHCl_3_-MeOH)	[8]
**15**	cuneatin (7-hydroxy-2′-methoxy-4′,5′-(methylendioxy)isoflavone)	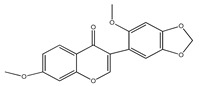	*E. polystachya*	Bark (CHCl_3_-MeOH)	[8]
**16**	7-hydroxy-2′,4′,5′-trimethoxyisoflavone	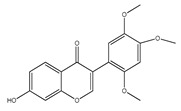	*E. polystachya*	Bark (CHCl_3_-MeOH) Heartwood (Aqueous)	[8]
**17**	(*α*-*R*)-*α*,3,4,2′,4′-pentahydroxydihydrochalcone	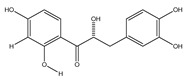	*E. polystachya*	Bark (CHCl_3_-MeOH)	[25]
**18**	(*αR*)-3′-C-*β*-D-xylopyranosyl-*α*,3,4,2′,4′-pentahydroxydihydrochalcone	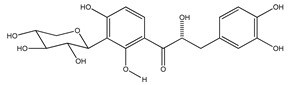	*E. polystachya*	Bark (CHCl_3_-MeOH)	[25]
**19**	(*αR*)-3′-*O*-*β*-D-xylopyranosyl-*α*,3,4,2′,4′-pentahydroxydihydrochalcone	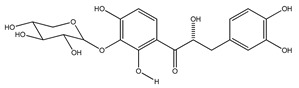	*E. polystachya*	Bark (CHCl_3_-MeOH)	[25]
**20**	4′,5,7-trihydroxy-8-methyl-6-(3-methyl-[2-butenyl])-(2*S*)-flavanone	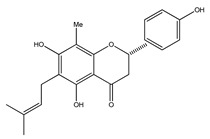	*E. texana*	Aerial parts (CH_2_Cl_2_-MeOH)	[26,27]
**21**	4′,5,7-trihydroxy-6-methyl-8-(3-methyl-[2-butenyl])-(2*S*)-flavanone	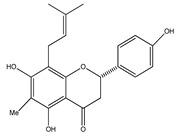	*E. texana*	Aerial parts (CH_2_Cl_2_-MeOH)	[26,27]
**22**	4′,5-dihydroxy-7-methoxy-6-(3-methyl-[2-butenyl])-(2*S*)-flavanone	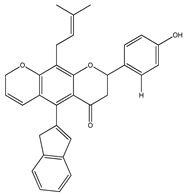	*E. texana*	Aerial parts (CH_2_Cl_2_-MeOH)	[26,27]
**23**	7-hydroxy-4′-methoxyisoflavone	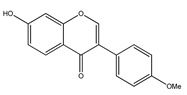	*E. polystachya*	Heartwood (Aqueous)	[28,29]
**24**	4′-*O*-methyl-8-prenylnaringenin	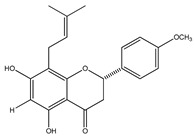	*E. platycarpa*	Leaves (Methanolic)	[30]
**25**	5,4′,1″-trihydroxy-6,7-(3″,3″-dimethylchroman)flavone	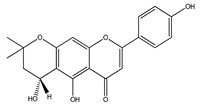	*E. platycarpa*	Branches (Methanolic)	[30]
**26**	(2*S*)-4′-*O*-methyl-6-methyl-8-prenylnaringenin	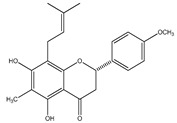	*E. platycarpa*	Branches and leaves (Methanolic)	[30]
**27**	5,7-dihydroxy-6-methyl-8-prenylflavanone	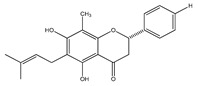	*E. platycarpa*	Branches and leaves (Methanolic)	[30]
**28**	5,7-dihydroxy-8-methyl-6-prenylflavanone	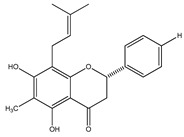	*E. platycarpa*	Branches and leaves (Methanolic)	[30]
**29**	5,7-dihydroxy-6-prenylflavanone	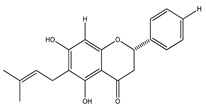	*E. platycarpa*	Branches (Methanolic)	[30]
**30**	5-hydroxy-7-methoxy-8-prenylflavanone	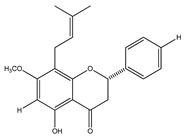	*E. platycarpa*	Leaves (Methanolic)	[30]
**31**	5,7-dihydroxy-8-prenylflavanone	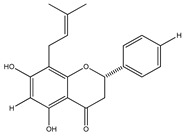	*E. platycarpa*	Branches (Methanolic)	[30]
**32**	subcoriacin (3-(2′-hydroxy-4′,5′-methylendioxyphenyl)-6-(3″-hydroxymethyl-4″-hydroxybut-2″-enyl)-7-hydroxycoumarin)	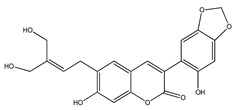	*E. subcoriacea*	Bark (Methanolic)	[31,32]
**33**	(+)-cathechin	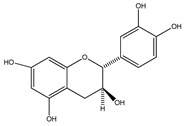	*E. platycarpa, E. subcoriacea*	Bark (Methanolic)	[30,31,33]
**34**	(−)-epicatechin	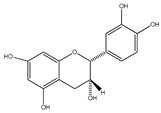	*E. platycarpa, E. subcoriacea*	Bark (Methanolic)	[30,31,33]
**35**	(+)-afzelechin	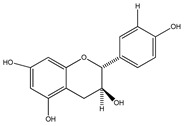	*E. platycarpa, E. subcoriacea*	Bark (Methanolic)	[30,31,33]
**36**	eriodictyol	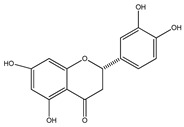	*E. platycarpa, E. subcoriacea*	Bark (Methanolic)	[30,31,33]
**37**	(+)-catechin-3-*O-β*-D-galactopyranoside	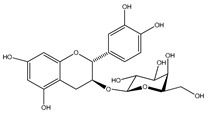	*E. platycarpa, E. subcoriacea*	Bark (Methanolic)	[30,31,33]
**38**	quercetin-3-*O-β*-D-galactopyranoside	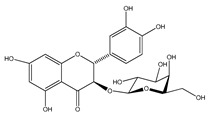	*E. platycarpa, E. subcoriacea*	Bark (Methanolic)	[30,31,33]
**39**	2′,4′-dihydroxychalcone-6′-*O-β*-D-glucopyranoside	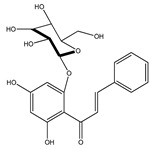	*E. polystachya*	Bark (Water/MeOH)	[34]
**40**	*α*,3,2′,4′-tetrahydroxy-4-methoxy-dihydrochalcone-3′-C-*β*-glucopyranosy-6′-*O*-*β*-D-glucopyranoside	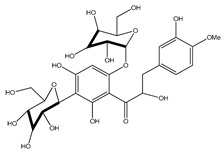	*E. polystachya*	Bark (Water/MeOH)	[34]
**41**	7-hydroxy-5,8′-dimethoxy-6′*α*-*L*-rhamnopyranosyl-8-(3-phenyl-transacryloyl)-1-benzopyran-2-one	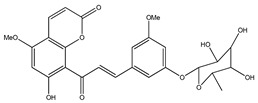	*E. polystachya*	Bark (Water/MeOH)	[34]
**42**	6′,7-dihydroxy-5,8-dimethoxy-8(3-phenyl-trans-acryloyl)-1-benzopyran-2-one	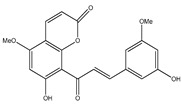	*E. polystachya*	Bark (Water/MeOH)	[34]
**43**	9-hydroxy-3,8-dimethoxy-4-prenylpterocarpan	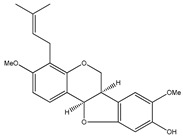	*E. polystachya*	Bark (Water/MeOH)	[34]
**44**	5,4′-dihydroxy-7,2′-dimethoxyl-isoflavone	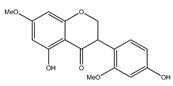	*E. polystachya*	Bark (Water/MeOH)	[34]
**45**	*α*,4,4′-trihydroxy-dihydrochalcone-2′-*O-β*-D-glucopyranoside	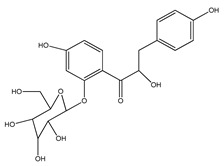	*E. polystachya*	Bark (Water/MeOH)	[34]
**46**	(3*R*)-5,7-2′,4′-tetrahydroxyl-3′-methoxyl-isoflavanone	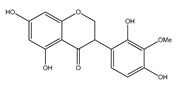	*E. polystachya*	Bark (Water/MeOH)	[34]
**47**	flemichapparin C (9-methoxy-2,3-methylenedioxycoumestan)	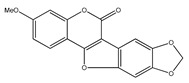	*E. polystachya*	Bark (Water/MeOH)	[34]
**48**	neohesperidin-dihydrochalcone	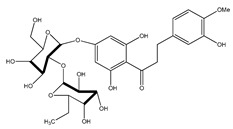	*E. polystachya*	Bark (Water/MeOH)	[34]
**49**	hesperetin dihydrochalcone glucoside	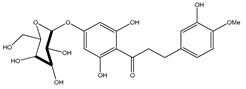	*E. polystachya*	Bark (Water/MeOH)	[34]
**50**	aspalathin	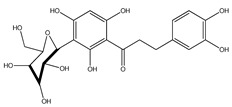	*E. polystachya*	Bark (Water/MeOH)	[34]
**51**	sandwicensin	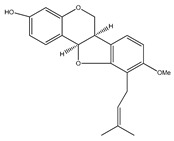	*E. polystachya*	Bark (Water/MeOH)	[34]
**52**	2′-*O-α*-L-rhamnopyranosyl- *α*,6′-dihydroxy-4′-acetyl-4-methoxydihydrochalcone	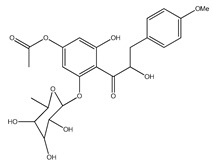	*E. polystachya*	Bark (Water/MeOH)	[35]
**53**	6′methoxy-sieboldin	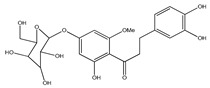	*E. polystachya*	Bark (Water/MeOH)	[35]
**54**	2′-*O-β*-D-glucopyranosyl-4′-methoxy-4-hydroxy-3-isoprenyldihydrochalcone	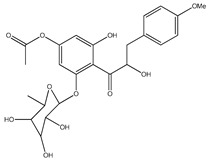	*E. polystachya*	Bark (Water/MeOH)	[35]
**55**	2′,4′,6′-trihydroxy-4,5-dimethoxy-3-isoprenyldihydrochalcone	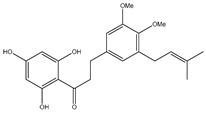	*E. polystachya*	Bark (Water/MeOH)	[35]
**56**	3′-C-*β*-glucopyranosyl-*α*, 2′,4′,6′-trihydroxy-4-methoxydihydrochalcone	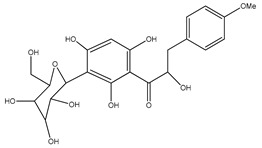	*E. polystachya*	Bark (Water/MeOH)	[35]
**57**	3′-C-*β*-glucopyranosyl-*α*, 2′,4-trihydroxy-4′,6′-dimethoxydihydrochalcone	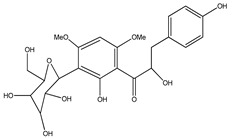	*E. polystachya*	Bark (Water/MeOH)	[35]
**58**	3-hydroxyphloretin-4′-*O-β*-D-glucopyranoside	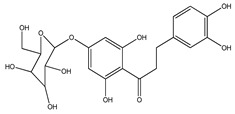	*E. polystachya*	Bark (Water/MeOH)	[35]
**59**	3,4′-dihydroxy-2,4,6-trimethoxydihydrochalcone	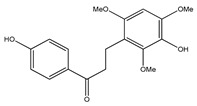	*E. polystachya*	Bark (Water/MeOH)	[35]
**60**	2′,4′,4-trihydroxy-3′-methoxydihydrochalcone	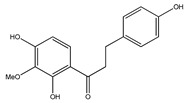	*E. polystachya*	Bark (Water/MeOH)	[35]
**61**	2′,4′,6′,4-tetrahydroxy-3,5-diisoprenyldihydrochalcone	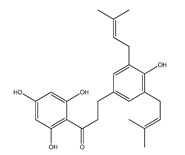	*E. polystachya*	Bark (Water/MeOH)	[35]
**62**	3′-C-*β*-glucopyranosyl-*α*,2′,4′,3,4-pentahydroxydihydroxychalcone	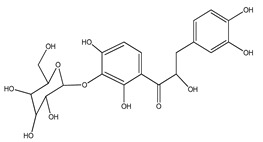	*E. polystachya*	Bark (Water/MeOH)	[35]
**63**	3′-*O-β*-D-glucopyranosyl-*α*,4,2′,4′,6′-pentahydroxydihydrochalcone	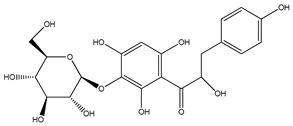	*E. polystachya*	Bark (Water/MeOH)	[36]
**Sterols and terpenoids**					
**64**	oleanolic acid	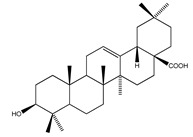	*E. platycarpa*	Branches (Methanolic)	[30]
**65**	*β*-sitosterol	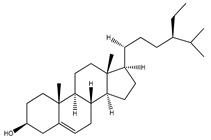	E. platycarpa	Branches (methanolic)	[30]
**66**	*β*-sitosteryl *β*-D-glucopyranoside	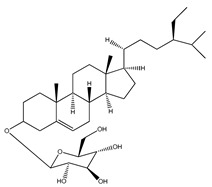	*E. platycarpa*	Branches (Methanolic)	[30]
**67**	*β*-sitosteryl palmitate	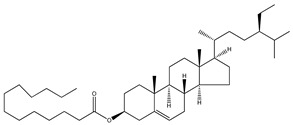	*E. platycarpa*	Branches (Methanolic)	[30]
**68**	stigmasterol	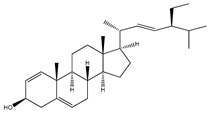	*E. polystachya*	Bark (CHCl_3_-MeOH)	[8]
**69**	3-*O*-acetyl-11*α*,12*α*-epoxy-oleanan-28,13*β*-olide	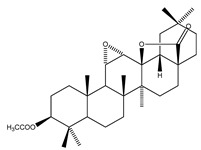	*E. platycarpa*	Branches (Methanolic)	[30,33]
**70**	lupeol	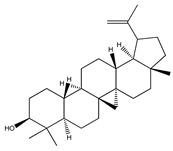	*E. platycarpa*	Branches (Methanolic)	[30]
**71**	betulinic acid	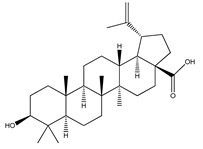	*E. platycarpa*	Branches (Methanolic)	[30]
**72**	3-*O*-acetyloleanolic acid	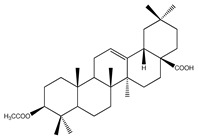	*E. platycarpa*	Branches and leaves (Methanolic)	[30]
**Fatty acids**					
**73**	stearic acid	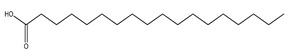	*E. polystachya*	Branches and leaves (Ethanolic)	[12]
**74**	arachidic acid	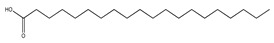	*E. polystachya*	Branches and leaves (Ethanolic)	[12]
**75**	palmitic acid	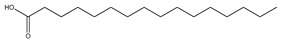	*E. polystachya*	Branches and leaves (Ethanolic)	[12]
**76**	linoleic acid	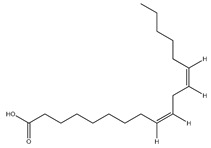	*E. polystachya*	Branches and leaves (Ethanolic)	[12]
**Hydroxybenzene**					
**77**	syringol	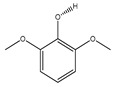	*E. polystachya*	Branches and leaves (Ethanolic)	[12]
**78**	catechol	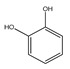	*E. polystachya*	Branches and leaves (Ethanolic)	[12]
**Cyclohexane**					
**79**	3-*O*-methyl-myo-inositol	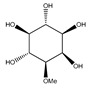	*E. platycarpa*	Branches (Methanolic)	[30]
**80**	D-pinitol	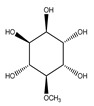	*E. polystachya*	Branches and leaves (Ethanolic)	[12]
**81**	lactic acid	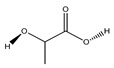	*E. polystachya*	Branches and leaves (Ethanolic)	[12]
**82**	2-furoic acid	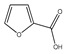	*E. polystachya*	Branches and leaves (Ethanolic)	[12]
**83**	succinic acid	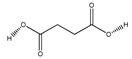	*E. polystachya*	Branches and leaves (Ethanolic)	[12]
**84**	fumaric acid	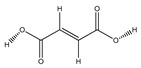	*E. polystachya*	Branches and leaves (Ethanolic)	[12]
**85**	sinapic acid	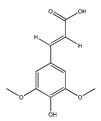	*E. polystachya*	Branches and leaves (Ethanolic)	[12]
**86**	N,N-diethyl carbamate	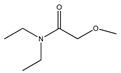	*E. polystachya*	Branches and leaves (Ethanolic)	[12]
**Carbohydrates or saccharides**					
**87**	D-erythrose	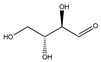	*E. polystachya*	Branches and leaves (Ethanolic)	[12]
**88**	D-mannose	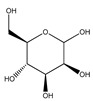	*E. polystachya*	Branches and leaves (Ethanolic)	[12]
**89**	D-arabinose	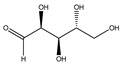	*E. polystachya*	Branches and leaves (Ethanolic)	[12]
**90**	xilose	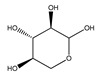	*E. polystachya*	Branches and leaves (Ethanolic)	[12]
**91**	trehalose	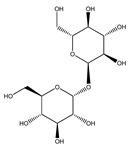	*E. polystachya*	Branches and leaves (Ethanolic)	[12]
